# Similar Sensitivity to Ladder Contours in Macular Degeneration Patients and Controls

**DOI:** 10.1371/journal.pone.0128119

**Published:** 2015-07-14

**Authors:** Andrew M. Haun, Eli Peli

**Affiliations:** 1 University of Wisconsin-Madison, Department of Psychology, Madison, Wisconsin, United States of America; 2 Schepens Eye Research Institute, Mass Eye and Ear, Department of Ophthalmology, Harvard Medical School, Boston, Massachusetts, United States of America; New York University, UNITED STATES

## Abstract

**Purpose:**

To determine whether people with central field loss (CFL) from macular degeneration have improved ability to recognize a particularly difficult spatial configuration embedded in noise, the peripherally-viewed ‘ladder contour’. The visibility of these configuration has been linked to general contour integration ability and crowding limitations in peripheral vision.

**Methods:**

We used a trial-based yes-no task. CFL patients and normally-sighted controls performed the task, looking for ladder contours embedded in a field of randomly oriented Gabor patches, at a range of stimulus presentation times (varying stimulus difficulty). Viewing eccentricity in CFL patients was set by their preferred retinal loci (PRLs) and matched artificially in the control group. The contours were presented so as to be tangent to the CFL region, given a patient’s PRL location.

**Results:**

CFL and normally-sighted groups performed similarly on the task. The only significant determinant of performance was the viewing eccentricity.

**Conclusions:**

CFL patients do not seem to develop any improved ability to recognize ladder contours with their parafoveal retina, which suggests that there is no underlying improvement in contour integration or reduction in crowding limitations in the region of the PRL despite extended daily use.

## Introduction

Macular degeneration deprives a large portion of primary visual cortex of input. Central field loss (CFL) usually occurs well after the critical phases of visual development, and it seems that the deafferented portion of visual cortex is not recruited by other parts of the visual system. There have been some reports of “reorganization” of deafferented visual cortex [1–5], but it is unclear whether or not this reorganization is an adaptive response by the visual system that yields some visual benefit, or simply a side effect of deafferentation [2,4,6]. However, in light of numerous findings in the field of perceptual learning, it seems that CFL patients, who typically come to rely on a particular region of still-functioning retina, the *preferred retinal locus* (PRL)[7], to explore and examine scenes, might be expected to gain some benefit from training of parafoveal (peripheral) retina that people with intact central vision do not receive[8–10]. Laboratory training of visual abilities in the periphery can yield improvements in performance, but there is only very weak evidence that daily visual experience brings any improvements in basic visual abilities near the PRL [11,12]. The question, then, is what visual task might reveal the possible benefits of early visual system reorganization in response to CFL.

Here, we report a study of peripheral detection (or more accurately recognition) of *ladder contours* in CFL patients. We chose this task because an earlier report showed that these stimuli are not recognized in the visual periphery [13]; a plausible explanation for this is that the visual integration mechanisms underlying *crowding* make ladder detection difficult or impossible in the periphery [13–16]. We replicated the original finding of May and Hess in normally-sighted observers (see S1 Fig, S1 Appendix), and then found that by increasing stimulus size and with careful instruction, CFL patients could learn to detect the ladder stimuli. However, the ability to detect these ladder contours was not peculiar to CFL patients—a group of normally-sighted control subjects performed similarly. The fact that larger ladder contours are visible/recognizable in the periphery is consistent with the crowding hypothesis, as increasing size will bring an object out of fixed-size (but eccentricity dependent) crowding zones. Our negative result can thus be interpreted as absence of support for the notion that persistent use of a (preferred) peripheral retinal locus results in significantly decreased crowding near the PRL.

## Methods

Subjects included 11 individuals with CFL (aged from 36 to 78, mean age 62, standard deviation 13.6) and 8 normally-sighted subjects (aged from 40 to 68, mean 58, standard deviation 11.4). All participants gave informed consent in written form to participate in the study; the study and the consent procedure were approved by the Schepens Eye Research Institute IRB (Mass Eye and Ear human subjects protocol 12-188H-2010-09) and the Declaration of Helsinki.

Patient etiologies were as listed in Table 1. The PRLs of the patients were estimated with a Nidek MP1 (Padova, Italy) fixation exam over 30 seconds. Patient visual acuity was estimated using an electronic letter chart, and was found to correlate strongly with estimated PRL eccentricity (R^2^ = 0.678, p < .001). The control subjects all had normal (or corrected to normal) acuity and no known visual impairments. Some of the subjects in each group had prior experience in psychophysical tasks, but none were expert observers.

**Table 1:** 

#	type	etio	dem	PRL ecc	loc	MAR	3000	1000	500	250
C1	CFL	AMD	67M	5°	inf	3.0	x	x	x	x
C2	CFL	dry AMD	78M	9°	left	4.0	x	x	x	x
C3	CFL	AMD	66M	3.5°	inf	5.0	3.15	2.36	2.57	1.69
C4	CFL	AMD	74M	2°	inf	2.5	2.01	1.91	x	x
C5	CFL	SG JMD	43F	9°	left	8.0	1.81	1.27	1.42	0.61
C6	CFL	AMD	70F	10°	sup	8.0	1.40	0.70	x	x
C7	CFL	SG JMD	47M	9°	inf	8.0	1.41	1.15	0.70	0.76
C8	CFL	SG JMD	70F	4.5°	inf	4.0	0.84	0.97	0.68	x
C9	CFL	c&r JMD	62M	12°	inf	15.0	0.77	0.58	x	x
C10	CFL	NC JMD	36M	3°	inf	3.0	x	1.25	1.72	x
C11	CFL	AMD	68F	8°	inf	6.3	-0.13	x	x	x
N1	NORM	x	66F	8°	inf	≤1	x	x	x	x
N2	NORM	x	40F	4°	inf	≤1	3.09	3.31	2.17	3.05
N3	NORM	x	63M	8°	inf	≤1	2.23	2.64	1.75	0.41
N4	NORM	x	66F	6°	inf	≤1	1.24	2.97	1.42	1.64
N5	NORM	x	68M	6°	left	≤1	1.57	2.07	1.43	1.59
N6	NORM	x	45M	8°	inf	≤1	1.15	1.54	0.65	1.21
N7	NORM	x	68M	4°	left	≤1	0.59	0.68	0.23	0.33
N8	NORM	x	50F	14°	inf	≤1	0.27	0.07	0.33	x

**AMD**: Age-related Macular Degeneration; **SG**: Stargardt's; **c&r**: cone and rod dystrophy; **NC**: North Carolina dystrophy. **PRL ecc** indicates estimated PRL eccentricity or, in normals, distance of the fixation target from the center of the contour display. **loc** indicates the location of the target in the visual field, relative to the fovea. **MAR**–minimum angle of resolution in arcminutes. The last four columns are d' values for performance in completed blocks of the experiment, the same data plotted in **Fig 2** x’s indicate inability to complete a block of trials for a given condition.

**Fig 1:**
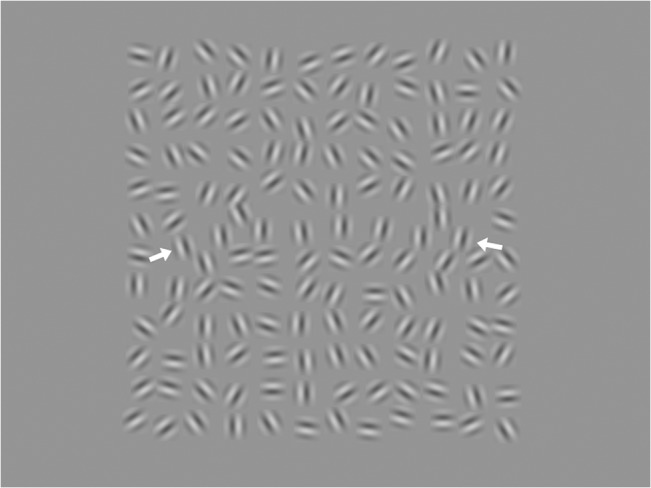
Target stimulus example. A 12x12 array of randomly oriented Gabor patches, eight of which are aligned along a curved path (ends marked here by white arrows). The aligned patches constitute a 'ladder contour', which is very difficult to perceive in peripheral vision. The image displayed here, including the gray surround, is the same (proportionally) as that shown on the experiment display.

Experiment stimuli were 12x12 arrays of randomly oriented Gabor patches, using the same stimulus parameters and construction algorithm as in May and Hess (2007)’s second experiment (allowing for contour curvature) [13]. We presented the arrays at a distance of about 50cm, so that the Gabor peak frequency was about 1.1 cycles per degree (4 times larger than the stimuli used in May & Hess 2007), and average separation between elements was 3 wavelengths (2.7 degrees). Gabors were presented at 100% contrast in the main experiment, and at 50% contrast in the version reported in S1 Appendix. The target for identification was a 'ladder contour', a smooth curved contour marked out by elements in the array with orientations orthogonal to the direction of the contour (Fig 1). Several presentation durations were used: 250ms, 500ms, 1000ms, and 3000ms, for the purposes of quickly training the CFL patients to do the task (in May & Hess's study, stimulus duration was 247ms). Before starting the task, a variable period of instruction was necessary to teach the CFL patients just what they were to be looking for in the experiment; this consisted of the experimenter pointing out a ladder contour in a static display, and allowing the patient to investigate the display until they understood the structure of the target. The normally-sighted controls were allowed to first foveate the contours, learning their appearance quickly, and then viewed stimuli peripherally. Instruction on the task only consisted of teaching the subjects, CFL and control, what they were looking for; we did not *train* any subjects on the task beyond a few practice trials so that the rhythm of stimulus presentation could be learned. Training may or may not be critical to measuring “effects” in this domain; in a replication of the original May and Hess study (pilot testing, as described in S1 Appendix), we found similar effects to those of May and Hess, despite most of those subjects being new to the task (and untrained, except for practice trials). Three of the CFL patients and two of the normal controls were unable to see the ladder contours (at their PRLs or viewed eccentrically, respectively).

We started with longer presentation times and worked down to the shorter; however, many of the CFL patients were unable to progress to the shortest presentation durations (250 msec), finding the timing too difficult to coordinate with their relatively unstable fixation patterns (according to their accounts of the difficulty). Except for one patient (C10), data collection began with a block of 3000ms trials, after an instruction and practice period, typically around 10–15 minutes, with untimed presentations (C10 began at 1000ms). Normally-sighted controls foveally fixated a spot at variable distance from the center of the Gabor array, and mostly succeeded in performing the task at the shortest presentation times; we distributed the fixation distances over control subjects so that the contours (when present) would fall at similar eccentricities to the CFL patients (mean patient PRL eccentricity: 6.8±3.3°; mean control eccentricity: 7.3±3.2°). Depending on whether a patient's PRL was left/right of or above/below the scotoma, vertical (for left/right PRLs; 3/11, 2 of whom could never see the contours) or horizontal (for above/below; 8/11 patients, one of whom could never see the contours) contours were presented. The controls fixated either below the Gabor arrays, looking for horizontal contours (7/9 subjects, 1 of whom could not see the contours, and 1 who had very low sensitivity) or to the left of the arrays, looking for vertical contours (two subjects, both of whom were able to do the task).

We used a yes-no experiment design to measure sensitivity to the ladder contours (frequently elderly naïve subjects have difficulty with a temporal 2AFC): half the trials (randomly selected) contained ladder contours embedded in an array of randomly oriented Gabors (Fig 1) while the other half consisted only of randomly oriented Gabor arrays. The subjects indicated verbally whether or not the stimulus had contained a contour, and the experimenter recorded their response. 100 trials were collected in separate blocks for each presentation duration. Patients and controls were asked before each trial–“Ready?”–until a steady present-response rhythm was attained; the experimenter periodically reminded the control subjects to maintain fixation, and asked them to report whether they accidentally glanced towards the target (these failures happened less than once per block, and those trials were repeated). Hit rate (H) and false alarm rate (FA) were measured for each observer and presentation time condition, and converted to the sensitivity measure *d'* = *z*(H)—*z*(FA), where *z* is the inverse of the cumulative normal distribution at *H* or *FA*. The display was a Trinitron CRT, running at 100 frames per second. The display mean luminance was 44 cd/m^2^. Gamma correction was performed through the video software and the experiments were written in Matlab (MathWorks, Natick MA), using Psychophysics Toolbox functions [17,18].

## Results

**Fig 2:**
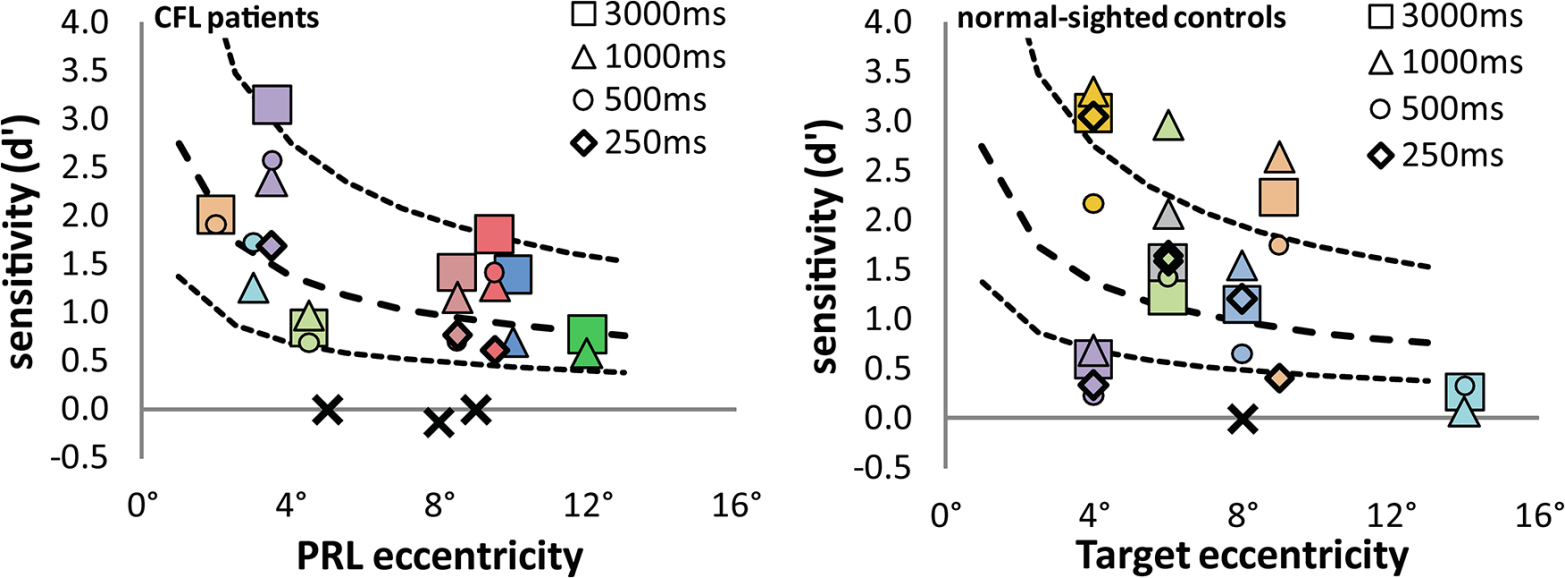
*Left panel*: CFL patient results, plotted as d' (signal-noise ratio) against estimated PRL eccentricity. Each color represents a different subject; different symbols are different presentation times as indicated in the legend. The X symbols are subjects who would not complete one block of trials (claiming to be unable to do the task), plus one patient (C11, the slightly negative d’ around 8°) who gamely completed 100 trials despite claiming not to know what the target looked like. The thick dashed line is a power function fitted to the data; the thin dashed lines are half and double the fitted line. *Right panel*: Normal control data, same conventions as for the CFL data. The lines are the same as in the first panel.

Results are as shown in Fig 2 The left panel shows, for all 11 CFL patients, *d'* as a function of contour eccentricity. Different symbols denote different presentation durations (which can be read as task difficulty), different colors to different individuals. The black 'X' symbols represent the PRL eccentricities of the patients who were unable to complete a single block of the experiment due to complete inability to recognize the targets.

There is a trend towards lower sensitivity at larger PRL eccentricities, represented by the thick dashed line, which is a power function fit to the patient data averaged over presentation duration (excluding the individuals who couldn't do the task). The thin dashed lines are half and double the mean trend, a range that encompasses all the between- and within-subject variation. The right panel shows the normal control data, with the same conventions as the patient data, but retaining the fit-to-patient lines to help with comparison of the two groups.

A statistical analysis of the data—ANCOVA with stimulus duration and participant group as discrete factors, and with eccentricity as a continuous factor, excluding the participants who could never see the targets (the black X's in Fig 2)—confirms that there is an effect of eccentricity (F(1,49) = 9.18, p = 0.0046). This is expected, since changes in acuity and crowding with increasing eccentricity would make a constant-size stimulus progressively more difficult. There was no significant effect of control/patient group (F(1,49) = 0.19, p = 0.67) or of test duration (F(3,47) = 0.19, p = 0.91). No factorial interaction approached significance.

## Discussion

We began this study with the hypothesis that cortical reorganization in CFL patients might enable them to recognize, with their peripheral PRLs, a spatial configuration—the ladder contour—that normally cannot be seen in the visual periphery. Our basic finding that increasing the size of the stimuli makes the ladder contours visible in the periphery is what would be predicted if inflexible crowding zones underlie ladder invisibility in the original task. In adapting the stimulus (making it bigger) to make the task possible for all the CFL patients, we obtained the same effect of visibility in normally-sighted controls. Our conclusion is that there appears to be no significant difference between CFL and normally-sighted individuals when it comes to perception of these difficult stimuli. This, combined with the likely common neural substrate underlying both the difficulty of seeing ladder contours and the phenomenon of crowding [13,16], suggests that CFL patients do not develop significantly smaller crowding zones near their PRLs.

A recent study by Chung [11] only partially confirms this view—she found that the extent of the crowding zone near the PRL was similar in CFL patients and normal controls, but only when measured tangentially to the scotoma boundary, similar to the arrangement of our contour stimuli. Chung found a small decrease in the size of the crowding zone when it was measured in the radial (to the fovea) direction, and derived an explanation for this decrease from a recent proposal that crowding zone shape is, at least partially, determined by eye movement patterns [11,19] which change as a consequence of CFL [20,21]. Since our target stimuli were embedded in 2-dimensional distractor arrays, radial interactions would also influence sensitivity to the ladder contours (crowding zones surround a feature like a contour) [13,16]; if there was a reduction in radial crowding in our patients, it was too small to be measured with the ladder contours.

The fact that we found no difference in performance on this task between the two groups does not mean there is no difference. Our normally-sighted controls did not only have normal retinas, but as a consequence they had different initial contact with the stimuli—i.e. the controls were allowed to foveally identify the ladder contours, a much easier task, before performing the difficult peripheral task. The CFL patients, meanwhile, had no such step-up, and had to bootstrap the task. The fact that, excepting their greater difficulty in seeing the brief presentations (which they routinely ascribed to fixational difficulties), the CFL patients performed similarly with the normally-sighted controls might indicate the presence of some other compensatory visual advantages. A negative result in this experiment also should not be interpreted as meaning that perceptual learning cannot occur near the PRL; learning can surely be induced by repeated practice on specific tasks designed to test individual psychophysical mechanisms. Our result means that, whatever learning has taken place in CFL patients near the PRL, it is either not at the level of contour integration mechanisms (i.e. crowding), or is of such small magnitude that we could not detect an effect.

## Supporting Information

S1 Appendix Replication of the finding by May and Hess (2007) that ladder contours are invisible in peripheral vision, while ‘snake’ contours (made up of collinear elements) are still visible. Subjects in this experiment were normally-sighted, and did not overlap with the control subjects in the main study. The important difference between the replicated study and the main study is in the size of the stimulus: when the stimulus is large enough, ladder contours are indeed visible in peripheral vision.
(DOC)

S1 Fig Box plots representing data from our replication of May & Hess 2007 (Experiment 2), and line plots of the average of May & Hess’s two observers for comparison (excluding their point at 6 degrees, where we did not collect data). Blue and red symbols are for snakes and ladders, respectively. We calculated d’ for the May & Hess data by treating the overall p(correct), which they reported, as the hit rate, and 1-p(correct) as the false alarm rate. This is an accurate means of d’ estimation if the response bias (1^st^ vs 2^nd^ interval) is not too large.
(TIF)
