# Supplementary material for: Similar Sensitivity to Ladder Contours in Macular Degeneration Patients and Controls
Source: PLoS One. 2015 Jul 14;10(7):e0128119. doi: 10.1371/journal.pone.0128119 (PMC4501758; doi:10.1371/journal.pone.0128119)
Supplement: S1 Appendix — Subjects in this experiment were normally-sighted, and did not overlap with the control subjects in the main study. The important difference between the replicated study and the main study is in the size of the stimulus: when the stimulus is large enough, ladder contours are indeed visible in peripheral vision. (DOC) [file pone.0128119.s001.doc]

**Appendix**

*Replication of May & Hess 2007*

Our reason for using ladder contours as target stimuli in the main experiment was that a previous study^13^ (May & Hess 2007, referred to by name from here on) found that visibility of ladders was negligible just a few degrees (of visual angle) from fixation. Before adapting the visual test to CFL patients, we first sought to replicate the original result. We gathered ten subjects (none of whom participated in the main experiment), between the ages of 21 and 40, and ran them through the same 2AFC ladder/snake detection task performed in the original study (May & Hess’s Experiment 2, with curved contours).

The stimuli were similar to those employed in the main study, but displayed at a smaller size and viewed from a greater distance. 'Snake' contours, whose elements are oriented parallel rather than perpendicular to the contour direction, were also included as test stimuli. In each block of trials, 50 snake and 50 ladder contour trials were randomly interleaved. Each trial consisted of two 250 msec stimulus presentations, separated by a 1000 msec ISI, one of which contained a contour (the target) and one of which contained only randomly oriented Gabors (the blank); the subject's task was to choose which interval (first or second) contained the contour. Separate blocks were run with the stimulus displays centered at 0, 4, and 8 degrees eccentricity. For the eccentric viewing conditions, a fixation point was continuously displayed on the screen; for the foveal viewing condition, the fixation point appeared in the center of the display between trials and during the ISI.

Each stimulus consisted of a 12x12 grid of Gabor patches, randomly oriented except when a contour was present. We followed the stimulus construction parameters used in May & Hess 2007. Each Gabor patch had a center spatial frequency of 4.6 cycles per degree, and the elements were separated by, on average, 3 wavelengths at this frequency (0.65 degrees). These were viewed monocularly by subjects at a distance of 140cm. We set stimuli only at horizontal eccentricities, and target contours were always globally vertical (the only difference between our and M&H’s stimuli; they allowed contours to run in any direction). Each subject completed 2 or 3 sessions of the experiment (i.e. 100 or 150 ladder trials and the same number of snake trials). 2AFC d’ values were computed for each stimulus eccentricity, and these are plotted in Figure S1.

The figure shows that our group of ten subjects had qualitatively similar performance to the two subjects in May & Hess 2007, although our subjects all had better performance (higher sensitivity) for the snake contours at all three eccentricities. The likely reason for this difference is that in the original experiment, global contour direction was not constrained, while we kept our target contours globally vertical (by constraining the average orientation of the elements), in anticipation of using constant-orientation stimuli in CFL patients (to keep the stimulus tangent to the scotoma edge). This would have reduced uncertainty about target position for our subjects, and this perhaps was enough to cancel whatever benefit may have been gained by having part of the contour extend further towards the fovea (which would have occurred in some trials had we not constrained contour direction).

These results are presented merely to support the original finding that ladder contours are invisible in the periphery, which was our original motivation for trying these stimuli with CFL patients. However, once we determined (in preliminary testing) that no CFL patient would see the contours with the original stimulus and task parameters, we adjusted these until we found measurable performance, which led to the study reported in the main text.
