# Supplementary figures and images for: Similar Sensitivity to Ladder Contours in Macular Degeneration Patients and Controls
Source: PLoS One. 2015 Jul 14;10(7):e0128119. doi: 10.1371/journal.pone.0128119 (PMC4501758; doi:10.1371/journal.pone.0128119)

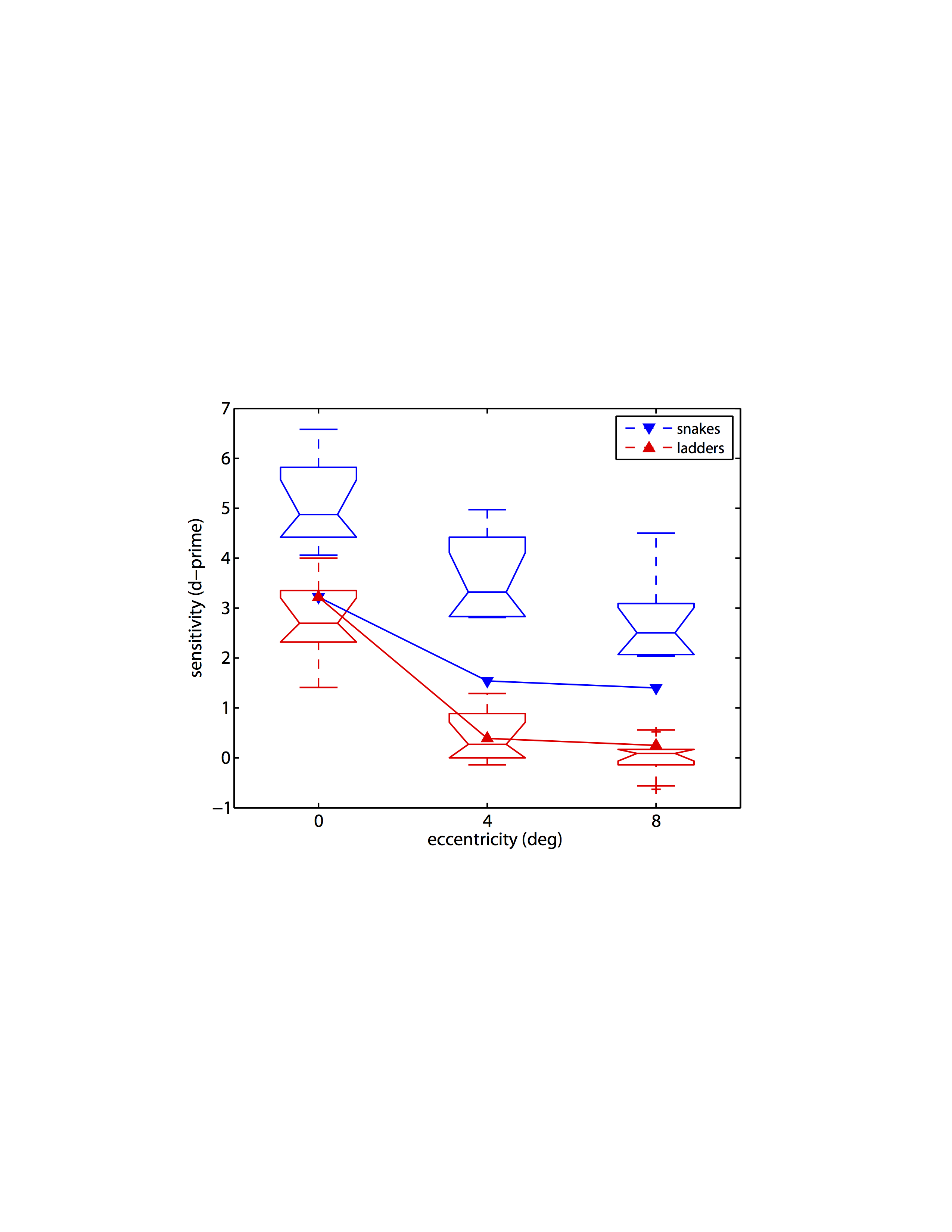

Supplement: S1 Fig — Blue and red symbols are for snakes and ladders, respectively. We calculated d’ for the May & Hess data by treating the overall p(correct), which they reported, as the hit rate, and 1-p(correct) as the false alarm rate. This is an accurate means of d’ estimation if the response bias (1st vs 2nd interval) is not too large. (TIF) [file pone.0128119.s002.tif]
